# GFRP Stiffened Plate with Square Cutout under Axial and Out-of-Plane Load

**DOI:** 10.3390/polym13081185

**Published:** 2021-04-07

**Authors:** Rahima Shabeen Sirajudeen, Alagusundaramoorthy P

**Affiliations:** 1 College of Engineering Guindy, Anna University, Chennai 600025, India; 2 Indian Institute of Technology Madras, Chennai 600036, India; asparao@gmail.com

**Keywords:** GFRP, composite, -stiffened plate, cutout, imperfection, buckling, axial load, out-of-plane load

## Abstract

The high-strength-to-weight ratio and corrosion resistance properties of glass-fiber-reinforced polymer (GFRP) composites makes them potentially well-suited for application in ship structures, bridges and off-shore oil platforms. These structures are often formed by stiffened plates and are subjected to axial load and out-of-plane load. Cutouts and openings are provided in the plates for access and maintenance. The main objective of this study was to examine the buckling behavior of GFRP-stiffened composite plates with square cutouts under a combination of axial and out-of-plane load up to failure. Four blade-stiffened composite plates without a cutout and four with square cutout were fabricated with stiffeners as a continuous layup of the flange plate using glass fiber and epoxy resin. The initial geometric imperfections were measured, and plate imperfections (Δx), stiffener imperfections (Δsy) and overall imperfections (Δsx) were calculated from the measurements. All fabricated-stiffened composite plates were tested up to failure. The finite element model was developed in ANSYS software and validated with the experimental results. It was observed that GFRP-stiffened composite plates failed by stiffener compression/stiffener tension mode of failure. The presence of out-of-plane loads and cutouts reduced the axial load carrying capacity of the stiffened composite plates.

## 1. Introduction

Stiffened panels are the main components of ship structures, bridge decks, off-shore oil platforms and aircraft structures. Stiffened plates are highly structurally efficient forms. The stiffeners are in the form of blade/flats, angles, T-sections and hat sections attached to one or both sides of the plate in longitudinal or in both longitudinal and transverse directions. The stiffeners add a little extra weight to the plates but increase the buckling and post-buckling strength of the plate enormously. Stiffened plates made of steel are susceptible to corrosion and incur high maintenance costs. Fiber-reinforced polymer (FRP) composites are a promising alternative to steel with lightweight, high-strength, corrosion resistance and inertness to environmental effects.

**Figure 1:**
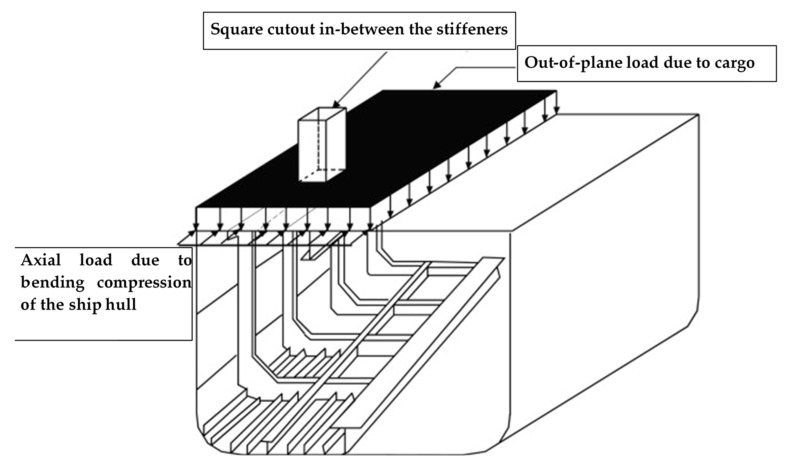
Typical ship deck composed of stiffened plates subjected to axial and out-of-plane load.

A-stiffened plate with application to ship structures (Figure 1) is considered for the present study. A typical ship deck is subjected to axial in-plane compression due to flexural bending of the ship hull. In addition, the ship deck is subjected to out-of-plane load due to cargo load. Further, ship decks are provided with openings or cutouts to provide access to the ship hull.

Carbon fiber-reinforced polymer (CFRP)-stiffened composite plates subjected to axial compression load was studied by Ishikawa et al. [1], Stevens et al. [2], Chiarelli et al. [3], Kong et al. [4], Falzon and Hitchings [5], Meeks et al. [6], Mo et al. [7], Zhu et al. [8], Feng et al. [9], Kolanu et al. [10], Massod et al. [11] and Tan et al. [12]. Glass fiber-reinforced polymer (GFRP)-stiffened composite plates subjected axial compression load was studied by Roberts et al. [13] and Kolanu et al. [14]. Broekel and Prusty [15] and Reinoso et al. [16] studied the behavior of stiffened composite panels under uniform transverse loading. The various authors have studied the buckling and post-buckling behavior of stiffened composite plates with a blade, I-shaped/T-shaped and hat stiffeners to understand the failure mechanism, quantify the reserve post-buckling strength, develop finite element models and formulate design methodologies. However, not many studies are available on the behavior of GFRP-stiffened composite plates under combined axial and out-of-plane loading.

Few researchers have studied the behavior of stiffened composite plates with holes. Nagendra et al. [17] investigated the behavior of blade-stiffened panels without and with a hole of size 0.1 times the distance between the stiffener under compression and shear. Falzon [18] investigated experimentally and analytically the post-buckling behavior of hat-stiffened CFRP composite panels with and without a centrally located circular hole of diameter 0.5 times the distance between the stiffeners. Alagusundaramoorthy and Shabeen [19] studied the strength of stiffened composite plates with square openings under out-of-plane load. Alagusundaramoorthy and Priyadarshini [20] and Anitha et al. [21] studied the behavior of GFRP-stiffened composite plates under axial and out-of-plane load with very large size cutouts. Sundaravadivelu et al. [22] compared the ultimate strength of steel and GFRP-stiffened composite plate. It is inferred that the presence of holes/cutouts influences the strength of stiffened composite plates. Studies on-stiffened composite plates with small cutouts/holes [17,18] or very large cutouts cutting across the intermediate stiffeners [20,21] are available. However, studies on cutouts extending between the full widths of the stiffener are very few.

The behavior of stiffened composite plates with cutouts and combined loading is complex and requires thorough investigation. In addition, the results available for steel-stiffened plates cannot be readily used for composite-stiffened plates due to inherent differences in properties of steel and composite.

Further, a dedicated study on-stiffened composite plates under combined axial and out-of-plane load and with cutout would be useful in ship structure applications. This is the motivation for the research. Based on this motivation, the objective is formulated.

This paper’s objective was to study the ultimate strength, load–deflection behavior, and failure mode of the blade-stiffened GFRP composite plates with square cutouts extending between the full width of the stiffener and with initial geometric imperfections under axial, out-of-plane and combined axial and out-of-plane loading. The scope of this study includes (i) fabrication of GFRP-stiffened composite plates with stiffeners as an integral part of the flange plate, (ii) measurement of initial geometric imperfections in the plate and stiffener, (iii) testing of GFRP-stiffened composite plates under axial load, out-of-plane loading and combination of axial and out-of-plane pressure load and (iv) prediction of the load-deflection behavior and failure mode using finite element analysis.

## 2. Test Specimens

Glass fiber-reinforced polymer (GFRP) plates-stiffened with blade-shaped stiffeners and square cutouts were considered for the experimental and analytical study. GFRP-stiffened composite plates without and with cutout were fabricated by hand layup process. Glass-fiber fabric woven roving mat (WRM) of area density 610 grams per square meter (gsm) and epoxy LY556 resin with HY951 hardener was used for the fabrication of GFRP-stiffened composite plates. Epoxy resin was used because of its superior mechanical properties when compared to vinyl ester and polyester resins. The properties of the resin and GFRP composite were found according to ASTM/ISO standards; the properties are tabulated in Table 1 and Table 2, respectively. The elastic modulus.

**Figure 2:**
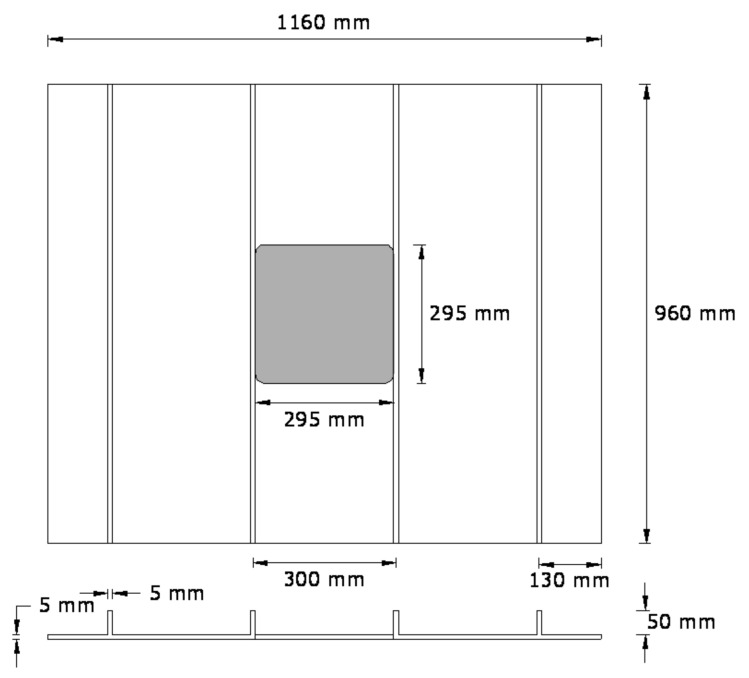
Geometry of glass-fiber-reinforced polymer (GFRP)-stiffened composite plate.

Rectangular GFRP-stiffened composite plates of length 960 mm, and width 1160 mm with four blade stiffeners at 300 mm center-to-center distance were fabricated. Two groups of GFRP-stiffened composite plates, namely, one group without cutout and the other group with square cutout, were fabricated. GFRP-stiffened composite plates with cutout were fabricated with a square cutout of size 295 mm × 295 mm, extending between the full widths of the stiffener. The geometry of the GFRP-stiffened composite plate is shown in Figure 2.

**Figure 3:**
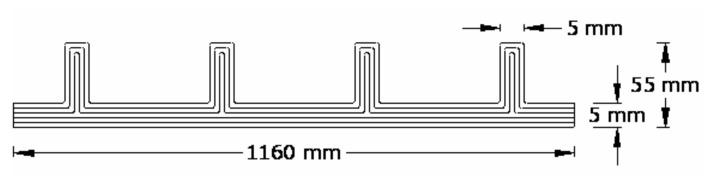
Layup structure of GFRP-stiffened composite plate (not to scale).

**Figure 4:**
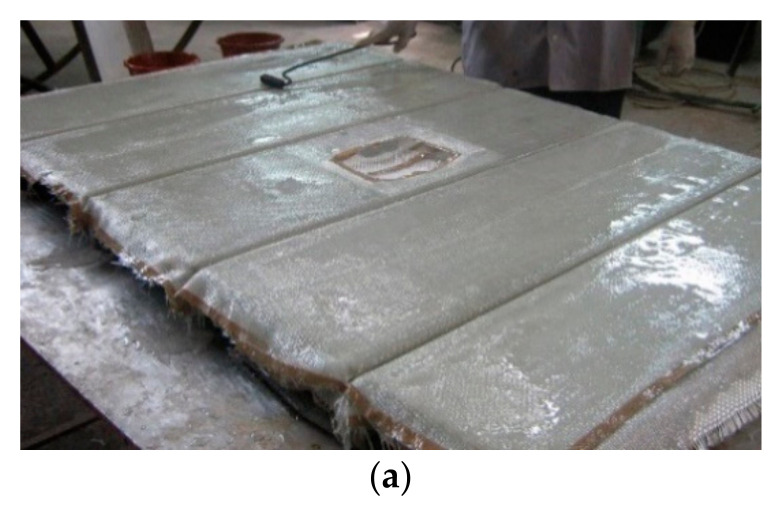

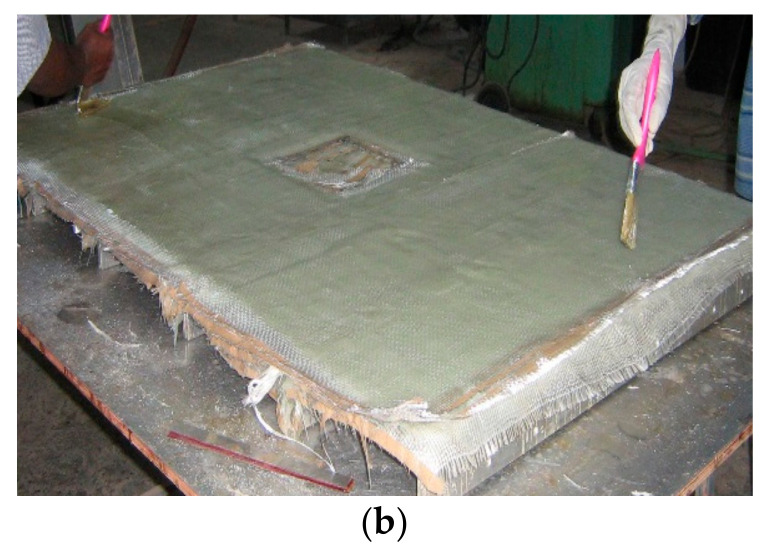
Casting of GFRP-stiffened composite plates. (**a**) Laying of the first three layers (**b**) Laying of last three layers.

**Figure 5:**
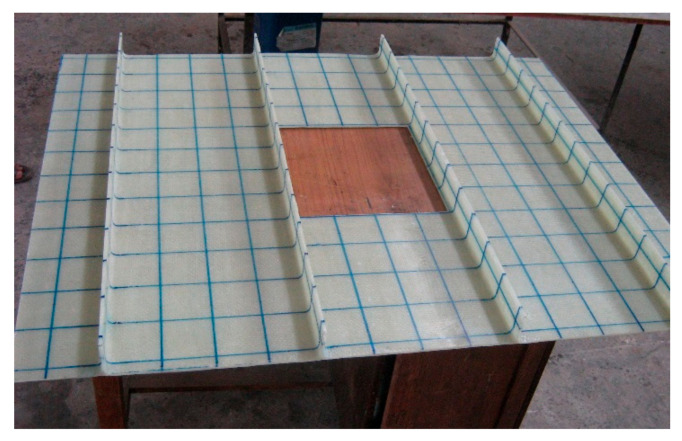
Fabricated GFRP-stiffened composite plate with cutout.

The layup consisted of 6 layers of woven roving mat, as shown in Figure 3. The first three layers were bent and made to pass through the stiffener (Figure 4a), and the next three layers were laid flat on the plate (Figure 4b). Thus, the stiffeners were cast integral with the plate. The specimens were cured at room temperature, and edges were trimmed. The specimens without cutout were designated as LSTP1, LSTP2, LSTP3 and LSTP4. Further, the specimens with cutout were designated as LSTPO1, LSTPO2, LSTPO3 and LSTPO4. Figure 5 shows a typical cast-stiffened GFRP composite plate with a cutout.

## 3. Initial Imperfections

Initial geometric imperfections on the top surface of the flange plate and on one side of the stiffener were measured at an interval of 75 mm in the longitudinal and transverse direction for all the cast specimens. Imperfections were also measured at the mid-height of the stiffener. Grids were marked for this purpose.

**Figure 6:**
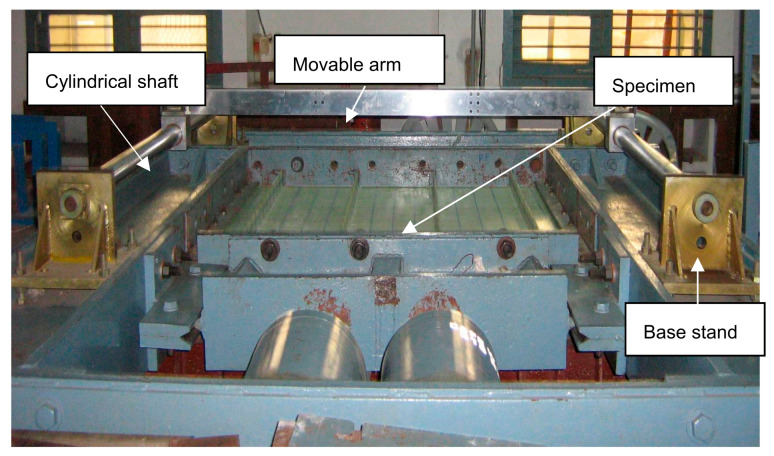
Initial imperfection measurement setup.

**Figure 7:**
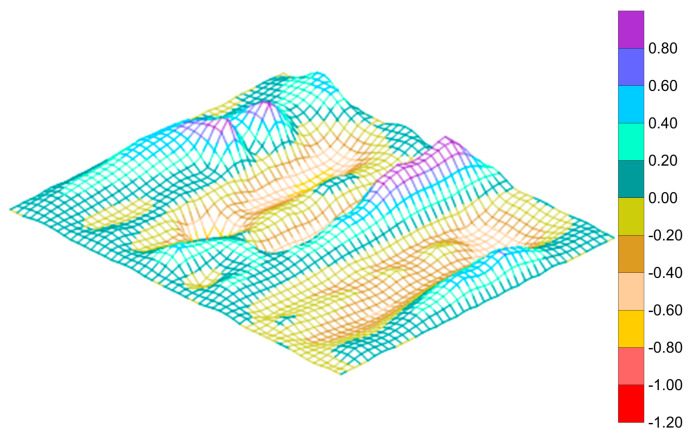
Imperfection profile of specimen LSTP1.

The imperfection measurement device consisted of a stainless-steel cylindrical shaft of 60 mm diameter and 1540 mm length connected to a base stand. A rectangular movable arm of size 1700 mm × 22 mm × 75 mm was connected between the two shafts and was capable of sliding along the longitudinal direction of the plate. The whole setup was rested on the loading frame, as shown in Figure 6. Dial gauges of accuracy 0.01 mm were attached to the movable arm and moved along the grid points. Imperfections were measured at 143 locations on the flange plate and 104 locations on the stiffener. The accuracy of the measurement was ensured by repeating the measurements until no changes in the measured values were observed. Isometric view of initial imperfections of the specimen LSTP1 is shown in Figure 7. The imperfection view of the other specimens is not shown in this paper.

The measured initial imperfections were converted into equivalent sinusoidal imperfections, namely plate imperfection (∆_x_), overall imperfection of the GFRP-stiffened composite plate (∆_sx_) and torsional imperfection of stiffeners (∆_sy_) according to the procedure by Alagusundaramoorthy et al. [23]. The calculated values are shown in Table 3.

## 4. Test Details

**Figure 8:**
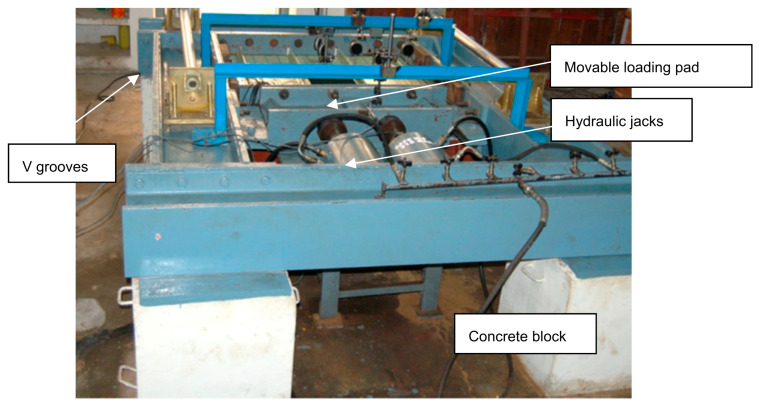
Test facility.

The GFRP-stiffened composite plates were tested under axial load, out-of-plane load and combined axial and out-of-plane load. A self-straining test rig available in the Ocean Engineering Department, Indian Institute of Technology Madras, was used for testing the GFRP-stiffened composite plates. The overall view of the test facility is shown in Figure 8.

The entire frame rested on four concrete blocks. Axial load was applied using two hydraulic jacks connected to a pump through a distributor. The capacity of each hydraulic jack was 1000 kN, and the length of the ram was 150 mm. The load from the two jacks was distributed uniformly across the cross-section of the specimen through the movable loading pad, which slides between the v-grooves along the longitudinal direction. Grooved plated were provided along the loading and reaction edges and simply supported boundary conditions were ensured along the four edges of the specimen. The supporting grooves at the edges of the plate restrained out-of-plane translational displacement and allowed rotation along the edges. The out-of-plane load was applied using inflatable airbags of capacity 400 kN/m^2^ and connected to a pneumatic compressor. The inflatable airbag of size 1080 mm × 880 mm × 140 mm was placed between the GFRP-stiffened composite plate and the rigid bottom grillage. An inflatable airbag with a square cutout at the center was fabricated to apply out-of-plane load uniformly on the test specimens with cutout.

**Figure 9:**
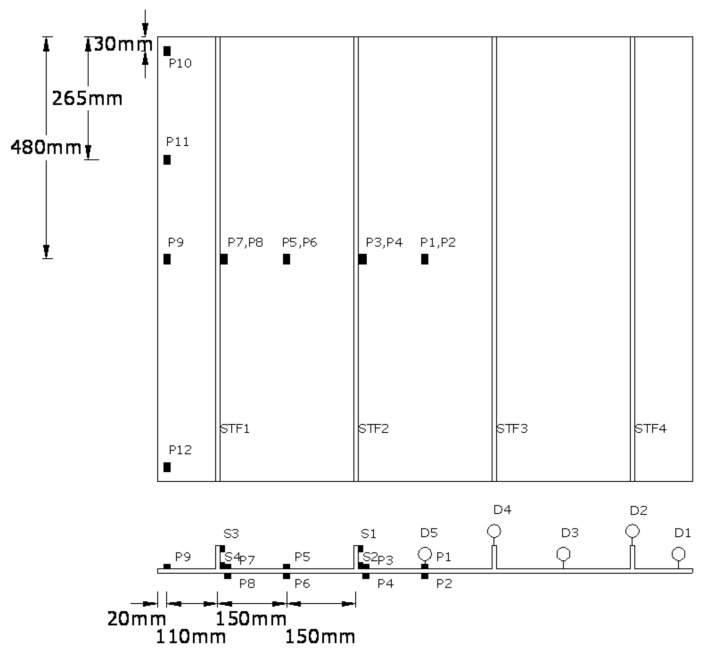
Position of dial and strain gauges. STF1, STF2, STF3, STF4—stiffeners. P1, P2…. P12—Position of strain gauges on flange plate. S1, S2, S3, S4—Position of strain gauges on stiffeners. D1, D2, D3, D4, D5—position of dial gauges.

The loading pattern of GFRP-stiffened composite plates is shown in Table 4. Out-of-plane deflection, axial deformation and the strains were measured, and the location of dial gauges and strain gauges, as shown in Figure 9. All specimens were tested until failure.

## 5. Finite Element Analysis

GFRP-stiffened composite plates were analyzed using ANSYS software. The plate and the stiffeners were modeled using the SHELL181 element. SHELL181 is a 4-noded shell element with finite strain and large rotation capability. All the layers had the same material properties, orientation and ply thickness. The thickness of each ply was taken as 0.84 mm. The GFRP-stiffened composite plate was defined as an orthotropic linear elastic material, and the properties were based on values obtained from material testing of GFRP (Table 2). Simply supported boundary conditions were ensured along all four edges. The measured geometric imperfections were introduced in the finite element model by defining the position of the nodes of the flange plate and stiffener according to the measured imperfection values. The nodes were connected by splines creating a geometrically imperfect surface. The plate and the stiffeners were connected by coincident nodes. Axial load was applied on the nodes along the loaded edge, and the out-of-plane load was applied uniformly over the flange plate surface. Optimum mesh with 3952 elements and an aspect ratio of 1 to 1.25 was generated based on the convergence study. The buckling analysis option was used to find the buckling load in FEA. Newton–Raphson method with arc-length control was used for the nonlinear post-buckling analysis. Nonlinear analysis was done with incremental axial load in the post-buckling range. The load was incremented in steps, and analysis was terminated when global instability was reached, which was found from the load/axial deformation graph.

## 6. Results and Discussions

### 6.1. Behavior of GFRP Stiffened Composite Plates under Axial Load

**Figure 10:**
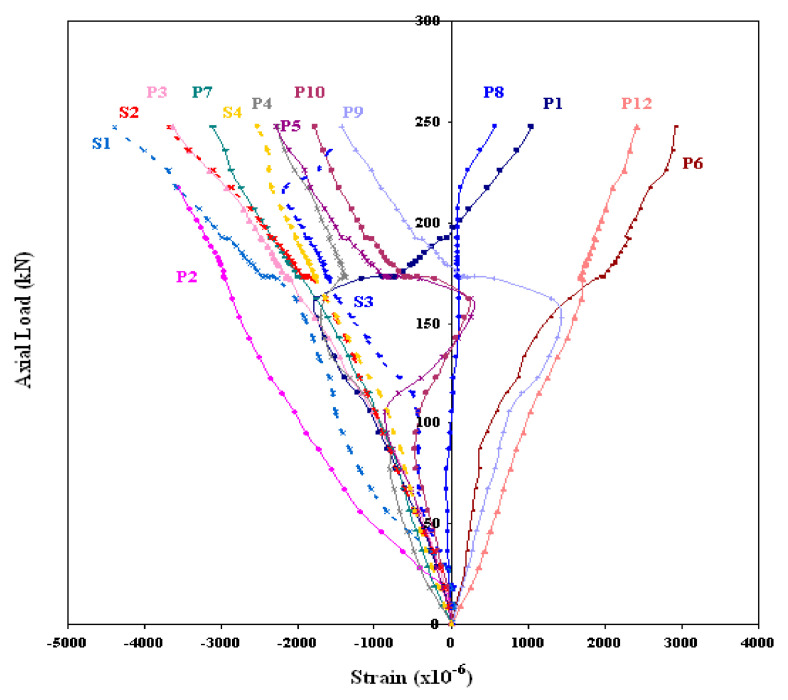
Load–strain behavior of LSTP1.

**Figure 11:**
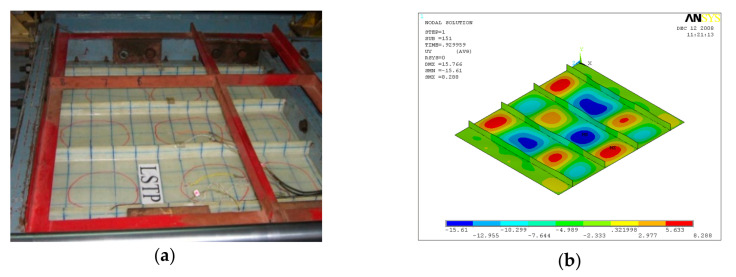
Buckling of LSTP1 (**a**) experiment (**b**) finite element analysis (FEA).

**Figure 12:**
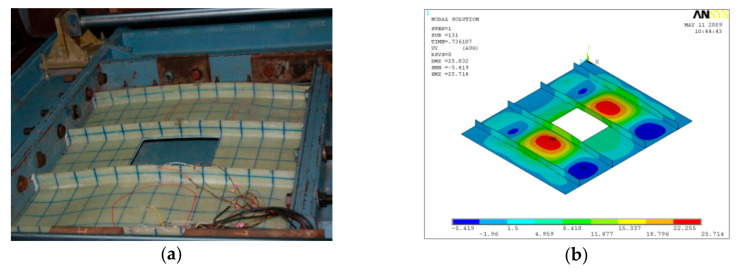
Buckling of LSTPO1 (**a**) Experiment (**b**) FEA.

GFRP-stiffened composite plates LSTP1 and LSTPO1 were tested up to failure under incremental axial load. Figure 10 shows the strain variation at various locations. At a load of about 170 kN, the strain values in plates P1, P5 and P9 experience a reversal in direction. This corresponded to the mode switching of the plate, as witnessed during the experiment. Initially, the flange plate between the stiffeners buckled into three half-sine waves, which switched to four half-sine waves (Figure 11) at a load of around 170 kN. The flange plate of LSTP1 between the stiffeners buckled into four half-sine waves (Figure 11). This was followed by progressive bending deformation of the stiffeners. Failure of one of the intermediate stiffeners (SFT2) was followed by failure of the other intermediate (STF3) and end stiffeners (STF4). Three of the four stiffeners failed (Figure 12).

Load/strain curves for the specimen LSTP1 indicate that the tip of the stiffener (SFT1) reached a maximum strain of 0.0044 than all other strain gauges. No failure was observed in the flange plate, and further, no debonding of stiffener from the flange plate was observed at failure.

**Figure 13:**
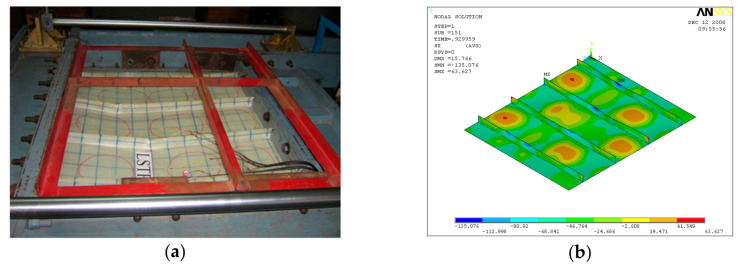
Failure of LSTP1 (**a**) Experiment (**b**) FEA.

Buckling of flange plate in-between the stiffeners was observed in GFRP-stiffened composite plate LSTPO1. The flange plate between the stiffeners STF1 and STF2, and STF3 and STF4 buckled into three half-sine waves, and the flange plate in between the stiffeners STF2 and STF3 with cutout did not show any distinct buckling mode (Figure 13). The failure of specimen LSTPO1 was initiated by buckling of the end stiffener (STF4), and the mode of failure was categorized as the stiffener-initiated failure. The buckling load was found to be 89 kN and 63 kN for LSTP1 and LSTPO1, respectively, from finite element buckling analysis. The buckling mode of LSP1 and LSTPO1 and failure pattern of LSTP1 obtained from finite element analysis (FEA) were compared with that of the experiment and are shown in Figure 11, Figure 12 and Figure 13, respectively.

### 6.2. Behavior of GFRP Stiffened Composite Plates under Out-of-Plane Load

**Figure 14:**
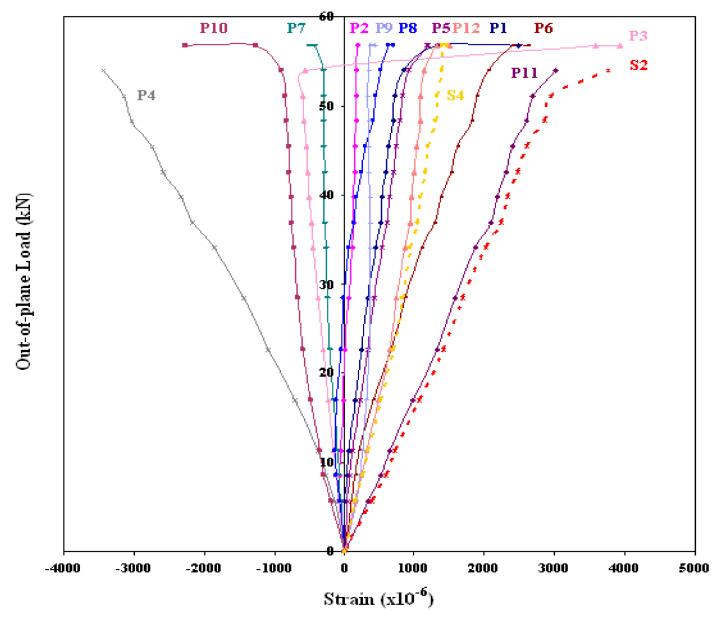
Load–strain behavior of LSTP4.

**Figure 15:**
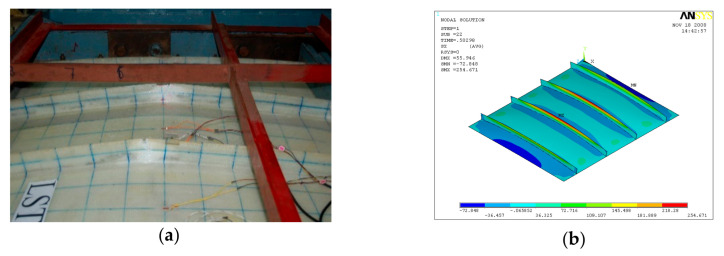
Failure of LSTP4. (**a**) Experiment (**b**) FEA.

**Figure 16:**
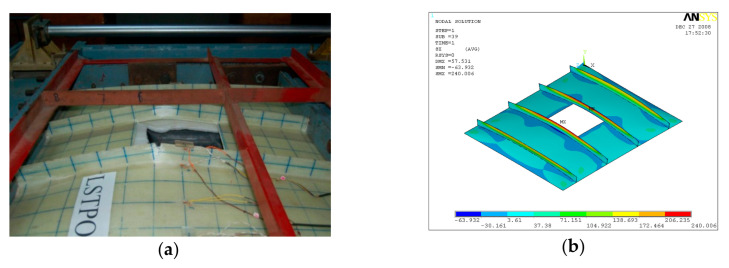
Failure of LSTPO4. (**a**) Experiment (**b**) FEA.

GFRP-stiffened composite plate LSTP4 and LSTOP4 were tested up to failure under incremental out-of-plane load. The uniform out-of-plane load causes overall bending with the tip of the stiffeners in tension. The GFRP-stiffened composite plate LSTP4 and LSTPO4 started load shedding at a load of 61 kN (55 kN/m^2^) and 56 kN (55 kN/m^2^), respectively and it was noted as the ultimate load. Figure 14 shows the strain variation at various locations of GFRP-stiffened composite plate LSTP4. Rupture of fabric at the tip of the stiffener was observed in the two intermediate stiffeners (STF2 and STF3) in both LSTP4 and LSTPO4, and the failure did not progress to the flange plate (Figure 15 and Figure 16). However, the strain gauges at the tip of the stiffener debonded before failure. Hence, the actual measurement of strain at the tip of the stiffener at failure could not be made. No damage was observed around the edges of the cutout. The stiffener did not debond from the flange plate at failure.

### 6.3. Behavior of GFRP Stiffened Composite Plates under Axial and Out-of-Plane Load

**Figure 17:**
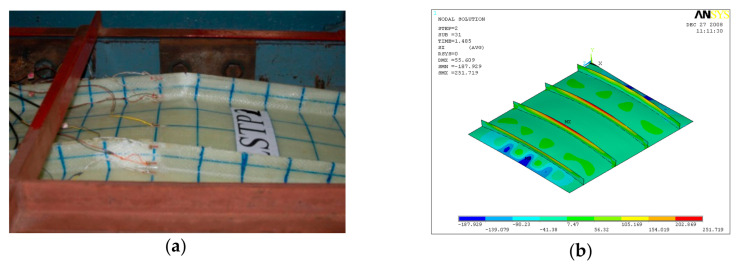
Failure of LSTP2. (**a**) Experiment (**b**) FEA.

**Figure 18:**
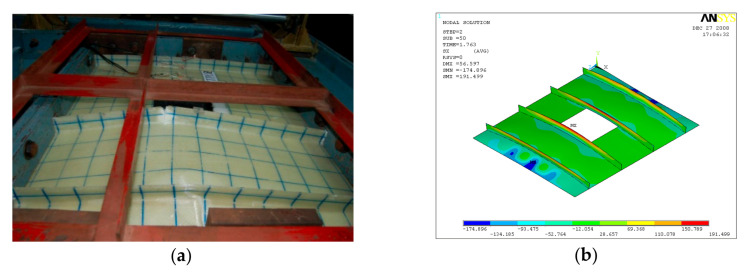
Failure of LSTPO2. (**a**) Experiment (**b**) FEA.

GFRP-stiffened composite plates LSTP2 and LSTPO2 were tested under a constant out-of-plane load of 22 kN (20 kN/m^2^) with an incremental axial load. The GFRP-stiffened composite plates were first subjected to an out-of-plane load of 22 kN. The out-of-plane load was maintained constant, and the axial load was progressively incremented to the failure of the GFRP-stiffened composite plates LSTP2 and LSTPO2. The out-of-plane load caused an overall bending of the GFRP-stiffened composite plate with the tip of the stiffeners in tension. The incremental axial load caused the marginal increase in mid-span out-of-plane deflection up to an axial load of 68 kN and 120 kN in LSTP2 and LSTPO2, respectively, and beyond which the out-of-plane deflection increased at a higher rate. Rupture of fabric was observed at the tip of the stiffeners (Figure 17 and Figure 18).

**Figure 19:**
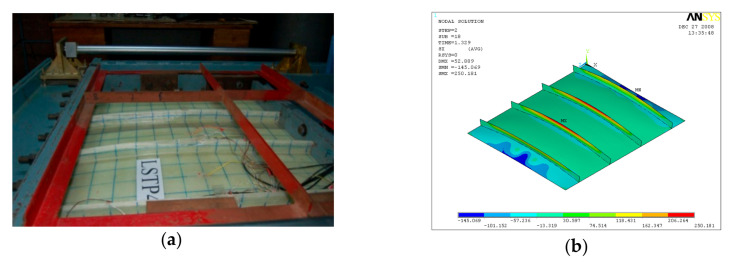
Failure of LSTP3. (**a**) Experiment (**b**) FEA.

**Figure 20:**
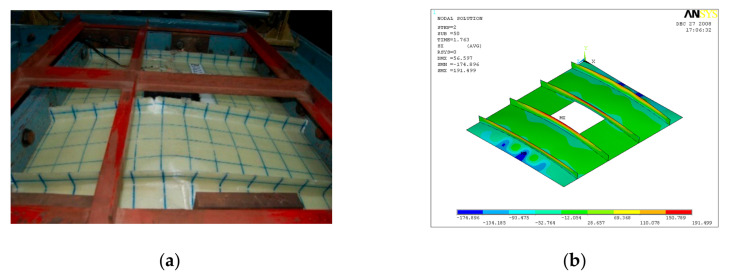
Failure of LSTPO3 (**a**) Experiment (**b**) FEA.

GFRP-stiffened composite plate LSTP3 and LSTPO3 were tested under a constant out-of-plane load of 44 kN (40 kN/m^2^). This applied out-of-plane load was maintained constant, and an incremental axial load was applied until the failure of the GFRP-stiffened composite plates. A rupture of the fabric was observed at the tip of the stiffeners of LSTP3 and LSTPO3 (Figure 19 and Figure 20).

**Figure 21:**
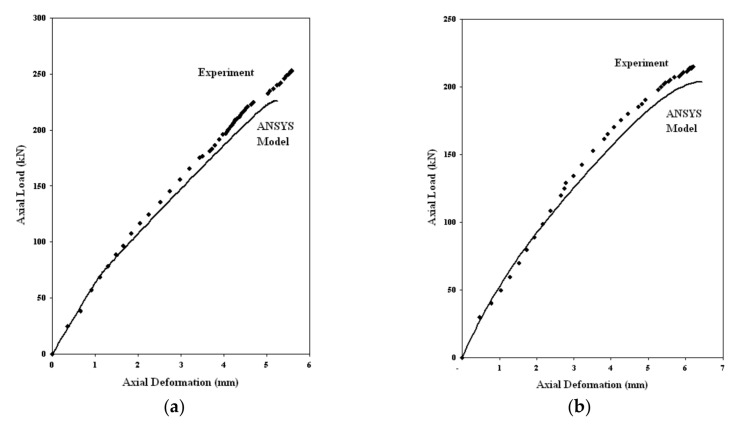

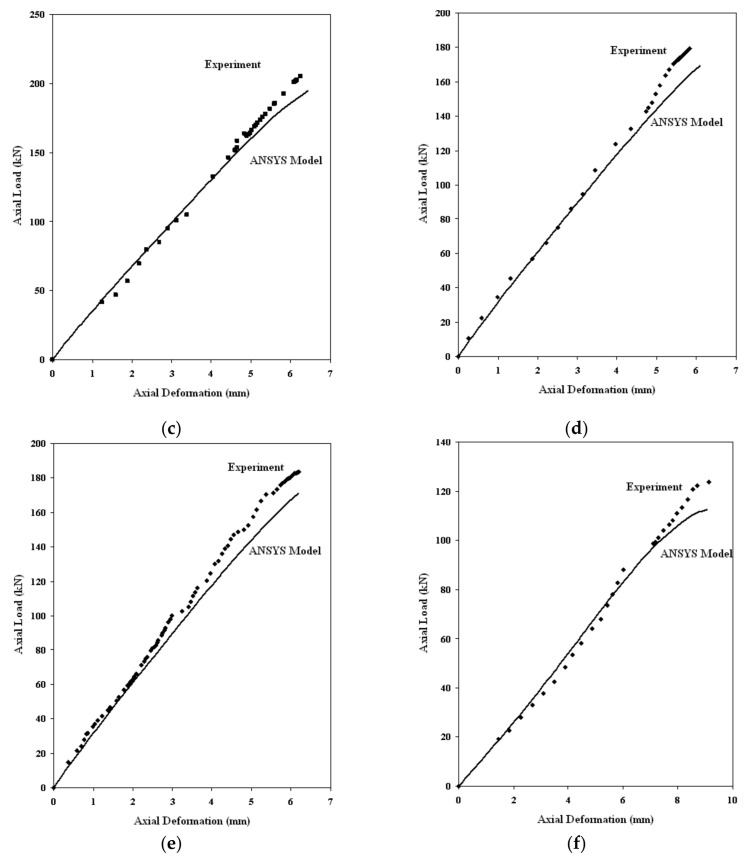
Axial load vs. deformation curve for GFRP-stiffened composite plates. (**a**) LSTP1 (**b**) LSTPO1 (**c**) LSTP2 (**d**)LSTPO2 (**e**) LSTP3 (**f**) LSTPO3.

**Figure 22:**
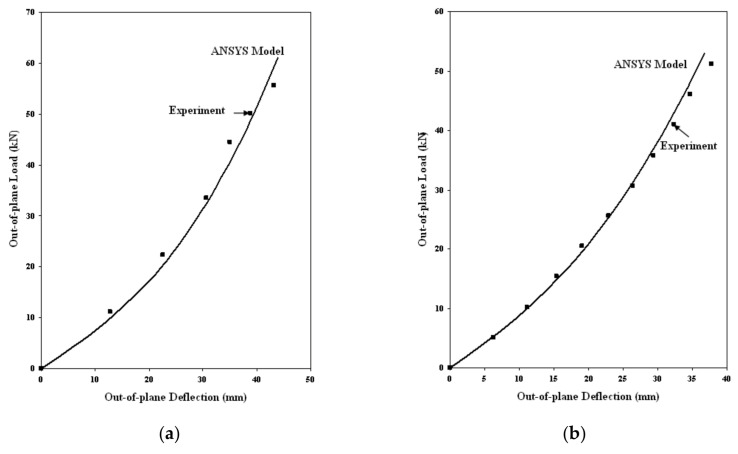
Out-of-plane load vs. deflection curve for GFRP-stiffened composite plates. (**a**) LSTP4 (**b**) LSTPO4.

Axial load vs. axial deformation curves for GFRP-stiffened composite plates subjected to axial load and combined axial and out-of-plane load are shown in Figure 21. Out of plane load vs. out of plane deflection curves for-stiffened composite plates subjected to only out-of-plane load is shown in Figure 22. The solid lines in Figure 21 and Figure 22 indicate the results from the finite element analysis in ANSYS, and the dots represent the data from the experiment. The slight variation in values between the experiment and analysis results, especially nearer the ultimate load, may be due to the assumptions regarding the material properties and boundary conditions assigned in the finite element model.

The ultimate axial loads and axial deformations for all the GFRP-stiffened composite plates obtained from the tests and FEA are given in Table 5. The FEA obtained axial loads and axial deformations were compared with that of experimental results. The statistical values of various parameters such as mean ($\bar{x}$), standard deviation (σ) and coefficient of variation (cv) were found to be within limits (Table 5), and hence, FEA values give a reliable estimation of loads and deformations. The location of failure of the specimens in FEA was determined from the stress contour. The LSTP1 and LSPO1 specimens failed by stiffener compression mode of failure, whereas all other GFRP-stiffened composite plates failed by stiffener tension mode of failure.

Table 6 shows the comparison of experimental and FEA out-of-plane ultimate load. The FEA underestimates the experimental out-of-plane load by 8%. Specimens LSTP4 and LSTPO4 failed by stiffener tension mode of failure. The failure mode was unaffected by the presence of cutout in GFRP-stiffened composite plates subjected to axial and out-of-plane loading. However, failure load was affected by the presence of cutout.

Table 7 shows the influence of square cutout on the axial load of the GFRP-stiffened composite plate. A decrease of 15% in axial load is observed in GFRP-stiffened composite plates subjected to zero and 1/3rd out-of-plane load. A decrease of 32% in axial load is observed in GFRP-stiffened composite plates subjected to 2/3rd out-of-plane load. Table 8 and Table 9 show the influence of out-of-plane load on the axial load carrying capacity of GFRP-stiffened composite plate without and with cutout, respectively. 1/3rd and 2/3rd out-of-plane loads decrease the axial load carrying capacity by about 16% and 28%, respectively, in GFRP-stiffened composite plates without cutout. 1/3rd and 2/3rd out-of-plane loads decrease the axial load carrying capacity by about 17% and 43%, respectively, in GFRP-stiffened composite plates with cutout. Initial application of out-of-plane load in GFRP-stiffened composite plates subjected to combined out-of-plane and axial load results in stiffener tension failure.

## 7. Conclusions

GFRP-stiffened composite plate without and with square cutout was fabricated and tested under combined axial load and out-of-plane pressure load. The GFRP-stiffened composite plates were also analyzed by the finite element method. Based on the experimental and finite element study on GFRP-stiffened composite plates with cutouts extending between the full widths of the stiffener, the following conclusions are made.

1. GFRP-stiffened composite plates with integral stiffeners could be made by hand layup process. This layup was observed to prevent the debonding of the stiffener from the flange plate;
2. The presence of the cutout reduced the ultimate axial load and ultimate out-of-plane load by 15% and 8%, respectively. However, the reduction in load-carrying capacity due to the presence of the cutout is maximum at 32% under combined axial and out-of-plane loads. Hence, it can be concluded that the presence of cutout is critical under combined axial and out-of-plane loads;
3. Stiffened composite plates with and without cutout fail by stiffener compression mode of failure under pure axial compression. Stiffened composite plates with and without cutout fail by stiffener tension mode of failure under out-of-plane load and under combined axial compression and out-of-plane load. Hence, it can be concluded that the presence of cutout does not change the mode of failure in stiffened composite plates;
4. Stiffened composite plates subjected to axial load undergo plate buckling between the stiffeners and fail by stiffener compression mode of failure. However, the stiffened composite plates subjected to an initial out-of-plane load followed by axial load undergo overall plate bending and fail by stiffener tension mode of failure. Hence, the initial application of out-of-plane load reduces the axial load carrying capacity and changes the failure mode of stiffened composite plates without and with cutouts;
5. The developed finite element analysis (FEA) model correlated well with the experimental results.

The experiment results and finite element analysis give an understanding of the behavior of stiffened composite plates with cutouts under combined axial and out-of-plane load. These results would help in the design of GFRP composite plates, especially for ship structure applications.

The lost strength due to cutouts could be improved by reinforcements around the cutout. This is the scope of future work.

## Figures and Tables

**Table 1:** Properties of resin.

Property	Value
Viscosity	9825 centipoises
Gel time of resin	36 min
Peak exothermic temperature	154 °C
Specific gravity	1.159
Tensile strength	42 MPa
Tensile modulus	3147 MPa
Flexural strength	95 MPa
Flexural modulus	3015 MPa
Water absorption (7 days)	0.2050%

**Table 2:** Properties of glass-fiber-reinforced polymer (GFRP).

Property	Value	Coefficient of Variation
Tensile strength—warp direction	250 MPa	3.06
Tensile strength—weft direction	211 MPa	8.53
Compressive strength	138 MPa	5.42
Shear strength	52 MPa	4.17
Flexural strength	384 MPa	9.91
Longitudinal modulus, (E_1_)	15,800 MPa	10.89
Transverse modulus, (E_2_)	15,333 MPa	9.96
Shear modulus (G_12_)	2806 MPa	1.19
Flexural modulus	15,388 MPa	5.07
Major Poisson’s ratio (ν_12_)	0.1386	3.28
Minor Poisson’s ratio (ν_21_)	0.1248	2.62

**Table 3:** Initial geometric imperfections.

Specimen	Measured Imperfections (mm)		
	Δ_x_	Δ_sx_	Δ_sy_
LSTP1	1.10	0.671	1.27
LSTP2	0.90	0.476	1.57
LSTP3	1.70	0.820	2.00
LSTP4	0.71	0.454	1.44
LSTPO1	1.22	1.83	1.81
LSTPO2	1.87	1.45	1.43
LSTPO3	1.89	1.37	1.51
LSTPO4	1.81	1.21	1.24

**Table 4:** Loading pattern of GFRP-stiffened composite.

Specimen	Specimen Type	Loading Pattern
LSTP1	Without cutout	Axial load until failure
LSTPO1	With cutout	Axial load until failure
LSTP2	Without cutout	1/3rd of ultimate out-of-plane load + Incremental axial load until failure
LSTPO2	With cutout	1/3rd of ultimate out-of-plane load + Incremental axial load until failure
LSTP3	Without cutout	2/3rd of ultimate out-of-plane load + Incremental axial load until failure
LSTPO3	With cutout	2/3rd of ultimate out-of-plane load + Incremental axial load until failure
LSTP4	Without cutout	Uniform out-of-plane loading until failure
LSTPO4	With cutout	Uniform out-of-plane loading until failure

**Table 5:** Axial ultimate loads were obtained from the experiment and FEA.

S. No.	Specimen	Out-of-Plane Load (kN/m^2^)	Axial Ultimate Load (kN)		Maximum Axial Deformation (mm)		$\frac{P_{\mathrm{EXPT}}}{P_{\mathrm{FEA}}}$	$\frac{{∆}_{\mathrm{EXPT}}}{{∆}_{\mathrm{FEA}}}$	Mode of Failure
			PEXPT	PFEA	ΔEXPT	ΔFEA			
1	LSTP1	0	249	226	5.58	5.24	0.907	0.939	SC ^1^
2	LSTP2	20	208	193	6.24	6.44	0.928	1.032	ST ^2^
3	LSTP3	40	180	171	6.44	6.18	0.950	0.960	ST ^2^
4	LSTPO1	0	211	204	6.20	6.67	0.967	1.075	SC ^1^
5	LSTPO2	20	176	169	5.82	6.08	0.960	1.045	ST ^2^
6	LSTPO3	40	121	112	9.11	9.03	0.926	0.991	ST ^2^
$\mathrm{Mean}\;\mathrm{value}\;(\bar{x}$ )							0.9397	1.007	
Standard deviation (σ)							0.0231	0.0525	
Coefficient of variation (cv)							0.0245	0.0521	

^1^ SC—stiffener compression mode of failure; ^2^ ST—stiffener tension mode of failure.

**Table 6:** Out-of-plane ultimate loads obtained from experiment and finite element analysis.

S. No.	Specimen	Out-of-Plane Ultimate Load (kN/m^2^)		$\frac{Q_{\mathrm{EXPT}}}{Q_{\mathrm{FEA}}}$	Reduction in Strength (%)	Mode of Failure
		QEXPT	QFEA			
1	LSTP4	61	56	0.914	-	ST ^2^
2	LSTPO4	56	52	0.929	8	ST ^2^

^2^ ST—stiffener tension mode of failure.

**Table 7:** Influence of square cutout on the ultimate axial load.

S. No.	Constant Out-of-Plane Load (q) (kN/m^2^)	Stiffened Plate without Cutout			Stiffened Plate with Cutout			Reduction in Strength (%)
		Specimen	Mode of Failure ^*^	P_EXPT_ (kN)	Specimen	Failure	P_EXPT_ (kN)	
1	0	LSPT1	SC	249	LSTPO1	SC	211	15%
2	20	LSTP2	ST	208	LSTPO2	ST	176	15%
3	40	LSTP3	ST	180	LSTPO3	ST	121	32%

* SC—stiffener compression mode of failure; ST—stiffener tension mode of failure.

**Table 8:** Influence of out-of-plane load on the axial load of GFRP-stiffened composite plates.

S. No.	Specimen	Constant Out-of-Plane Load (q) (kN/m^2^)	Axial Ultimate Load (P_EXPT_) (kN)	Reduction in Strength	Mode of Failure
1	LSPT1	0	249	-	SC
2	LSTP2	20	208	16%	ST
3	LSTP3	40	180	28%	ST

**Table 9:** Influence of out-of-plane load on the axial load of GFRP-stiffened composite plates with cutout.

S. No.	Specimen	Constant Out-of-Plane Load (q) (kN/m^2^)	Axial Ultimate Load (P_EXPT_) (kN)	Reduction in Strength	Mode of Failure
1	LSPTO1	0	211	-	SC
2	LSTPO2	20	176	17%	ST
3	LSTPO3	40	121	43%	ST
